# Experimental Characterization Framework for SLA Additive Manufacturing Materials

**DOI:** 10.3390/polym13071147

**Published:** 2021-04-02

**Authors:** Jordi Martín-Montal, Jesus Pernas-Sánchez, David Varas

**Affiliations:** Department of Continuum Mechanics and Structural Analysis, University Carlos III of Madrid, Leganés, 28911 Madrid, Spain; mjordi@ing.uc3m.es (J.M.-M.); dvaras@ing.uc3m.es (D.V.)

**Keywords:** additive manufacturing, stereolithography manufacturing, polymers (durable resin), 3D printing, mechanical behavior and characterization, printing parameters, dynamic regime

## Abstract

Additive manufacturing (AM) is driving a change in the industry not only regarding prototyping but due to the ease of including printed parts in final designs. Engineers and designers can go deeper into optimization and improvements of their designs without drawbacks of long manufacturing times. However, some drawbacks such as the limited available materials or uncertainty about mechanical properties and anisotropic behavior of 3D printed parts prevent use in large-scale production. To gain knowledge and confidence about printed materials it is necessary to know how they behave under different stress states and strain-rate regimes, and how some of the printing parameters may affect them. The present work proposes an experimental methodology framework to study and characterize materials printed by stereolithography (SLA) to clarify certain aspects that must be taken into account to broaden the use of this kind of material. To this end, tensile and compression tests at different strain rates were carried out. To study the influence of certain printing parameters on the printed material behavior, samples with different printing angles (θ = [0–90]) and different printing resolution (layer height of 50 and 100 µm) were tested. In addition, the effects of curing time and temperature were also studied. The testing specimens were manufactured in the non-professional SLA machine *Form 2* from Formlabs^®^ using resin called *Durable*. Nevertheless, the proposed experimental methodology could be extended to any other resin.

## 1. Introduction

Additive manufacturing (AM) is driving a change in industry; designers, manufacturers, logistics and many others are changing some of their procedures to adapt them to this manufacturing process. Industry lead times can be drastically decreased in prototyping; prior to AM development a prototype should passed through several steps before arriving at the designer; these steps cut through the different AM approaches and materials. This reduction allows engineers and designers to go deeper with optimization and improvement of their designs without drawbacks in lead times [1]. The impact of AM is not restricted to prototyping. Presently, several industries are introducing parts obtained through AM in final designs [2]; however, most of them are AM metal parts. The limited availability of materials, lower mechanical properties and anisotropic behavior of 3D printed parts limit the use in large-scale production. In addition, recently AM has demonstrated its ability to address major social challenges such as the emergency manufacture of equipment to combat COVID-19 during the pandemic. AM has been an additional manufacturing process that has met the huge demand for medical equipment and eased health disaster worldwide [3].

The aforementioned drawbacks have been tackled by employing different approaches. The development of different AM processes such as Fused Deposition Manufacturing (FDM), Selective Laser Sintering (SLS) or Stereolithography (SLA) open the industry to several material portfolios, such as plastics, metals or ceramics [4]. Mechanical performance has been improved using different reinforcements such as carbon fibers, particles, nanomaterials [5,6,7,8] or even using high-performance polymers [9]. Several studies have been done focusing on the tailoring of properties using AM, exploring the use of the material anisotropy as an advantage for mechanical or fracture performance [10,11,12,13].

Although SLA is one of the oldest AM methods, most articles focus on the mechanical properties of FDM parts, probably promoted by its rapid development and its cost. One of the drawbacks of FDM is its lack of surface accuracy and the presence of internal voids [14]. This is because the manufacturing process of FDM has the physical limit of the nozzle diameter, which is much lower in the case of SLA because the laser set of this size. Regarding the mechanical performance of the AM Materials, Chacon et al. [15] studied the effect of different FDM manufacturing parameters, such as layer height, feed rate or orientation, in the mechanical performance of PLA material. The authors concluded that some of those parameters influence the performance, increasing or decreasing the ductility or the strength. Similar results on raw material ABS have been obtained by Ryder et al. [16], in which the strength and the ductility depends on the orientation of the layers. However, all the results are done in quasistatic regime and only in tensile and flexural tests. Garzon-Hernandez et al. [17] using a similar FDM method focus on the influence of the number of layers in the mechanical performance proposing a constitutive modeling for the thermoplastic used. The authors concluded that the layer height decreased the strength, due to the increase of voids inherent of the FDM printing process [14]. Li et al. [13] focused on the dependence of the material printing orientation in the mechanical behavior of the printed specimens. The authors performed tensile and compression test on SLA resins under quasistatic regime and found different behavior for samples printed in the load direction. Finally, they take advantage of this behavior to produce optimized printing orientations for final parts. Hossain et al. [18] studied the behavior of a printed material obtained by digital light projector DLP process, focusing on the viscoelastic constitutive behaviour at low strain rates. However, the author does not focus on the influence of the printing parameters in the behaviour of the material. Miedzińska [19] performed quasistatic and dynamic (Split Hopkinson pressure bar) compression tests of raw SLA resin to study the strain rate dependence of SLA materials. The results shown an important dependence of the mechanical properties on the strain rate, but nevertheless the work does not study the effect of different manufacturing parameters of this AM technique on the mechanical performance. Hence it is clear that there is not an assessment program which gives researchers an experimental umbrella to unveil the driven parameters in the mechanical behavior of AM materials.

To get from prototype parts, focused on shape, to real functional parts it is necessary to know how the 3D material behaves under different stress states and strain rates regimes. Therefore, a complete assessment program which could provide engineers the knowledge about the material behavior would be very valuable. The aim of this research is to propose a “simple” experimental methodology or campaign capable of identifying the main behavior of the printed material to be used in real applications. It is considered that the experimental campaign should take into account the following situations:

- Different stress states: in real applications the AM parts can be subjected to a complex stress state.
- Loading and unloading: the AM parts can be subjected to transient stresses and hence it is necessary to know how they behave under load–unload cycles.
- Dynamic regime: to use AM parts for dynamic applications or protection under impulsive loads it is necessary to understand the behavior under such dynamic conditions.

In addition, it is necessary to take into account the influence of different manufacturing parameters of the AM techniques in the aforementioned conditions. For this approach, the stereolithography (SLA) manufacturing technique will be used due to its potential application to plastics and ceramics. The main driven parameters identified for this AM process and which will be considered are:

- Printing orientation
- AM layer height
- Post-curing process

## 2. Materials and Printing Methodology

The present work proposes an experimental methodology framework to study and characterize materials printed by stereolithography (SLA) to clarify certain aspects that must be taken into account to broaden the use of this kind of material. The SLA printing methodology consists of an additive type of manufacturing that uses a laser source projected over a platform to obtain the desired specimens. This beam of light goes across a liquid tank filled with a polymeric resin with photo-initiators and produces the selective curing in the surface areas hit by the ray trajectory. The curing process is done in the platform plane following a layer-by-layer building where the specimen is sustained by supports that aid the correct growth of the sample and its attachment to the building bed. The resolution of the printing not only comes defined by the laser diameter but also by the increment of the vertical displacement between each layer that can be selected by the user. In stereolithography-based current commercial printers, the aforementioned resolution runs in the range from hundreds of micrometers up to dozens.

The non-professional SLA machine *Form 2* from Formlabs^®^ has been used to print all the specimens of this work. To obtain a final printed specimen, several steps and devices are necessary. The printing process may lead to the obtaining of different mechanical properties and hence influencing the behaviour of a specimen. To identify which parameters may affect the final mechanical behaviour it is necessary to understand the whole process of the SLA printing and how the different steps may contribute to the final material properties. To obtain a printed specimen, first it is necessary to create an Standard Tessellation Language (STL) geometry file to afterwards define (according to the possibilities of the printer) all the settings, parameters and conditions to be considered during the printing process. Choosing different sorts of printing parameters is one of the major if not the most important part of all printing stages (since all subsequent stages will be conditioned by this). Components to be printed will be oriented in a certain position to enhance the laser-curing process, which could affect the final mechanical properties. In addition, scaffolding choice is also critical to ensure a proper sticking, guidance and support to the main piece without compromising the integrity, appearance and usability of the final specimens (especially those with a small cross-sectional area and which can be similar to the dimensions of the supports).

During the printing process, the precursor material is stored in liquid phase and heated in a tank located in the printing area laying above the set of lenses and mirrors. The printer uses a high-powered ultra-violet (UV) laser to selectively polymerize the UV-curable resin on a layer-by-layer basis. The vertical-layer resolution ranges from 50 to 100 µm controlled by the leading screws responsible for moving the platform in the vertical axis. This layer resolution could affect the specimen behaviour. When the printing process is completed, the specimen is introduced into an isopropanol solution bath to separate the remaining non-cured traces of resin from the sample. Further on, the sample may be post-cured by a heating ultraviolet device that provides temperature and UV radiation to harden the specimen by increasing the degree of polymerization and generate polymer chains cross-linking. Thus, the mechanical properties of the specimen and hence its mechanical behaviour could be modified by switching the parameters in post-curing stage leading to a large margin of maneuver and possibilities. Resin suppliers commonly give guidelines about this. Under the awareness that different steps during the manufacturing process can affect to the final mechanical behaviour of the specimen, three main parameters will be considered to be studied: the printing orientation, the AM layer height and the post-curing process.

It is worth mentioning that SLA printing technique has been chosen for this work because the specimens to be manufactured will have a higher quality compared with the ones obtained by other methodologies and techniques developed so far, such as FDM. The finest accuracy of the laser enables the design and creation of complex, thin, angled geometries in a versatile and fast way. The quality and reproducibility of the final product is substantially remarkable. The material chosen to develop the present study is a photopolymerizable resin that can be used to manufacture different specimens by stereolithography (SLA) in the non-professional SLA machine *Form 2* from Formlabs^®^. Form 2 is a stereolithography-based technology printer containing one 250 mW laser source with a 140 microns laser spot size. The laser is a 405 nm wavelength class 1 laser product by EN 60825-1:2007. The resin is called *Durable* and it is commonly used to prototype parts that would be made from polypropilene (PP). The durable resin is a proprietary mixture of methacrylated monomers and oligomers with a photoinitiator. The manufacturer does not share the specific composition with their users [20,21]. Once the part is printed, the Stereolithography Post-Cure Chamber of Formlabs called “Form Cure” was used. The curing process is carried out by 13 multi-directional LEDs with a 39 W power and 405 nm wavelength that can reach a highest curing temperature of 80 ${}^{\circ }$C. Nevertheless, the experimental methodology proposed in this work to study and characterize materials printed by stereolithography (SLA) could be extended to any other resin.

## 3. Experimental Framework and Test Setups

The present work proposes an experimental framework to help in the understanding of a printed material behaviour, and hence contributing to broaden its use. To characterize a material printed by stereolithography (SLA) at different stress states as well as to clarify how certain parameters may affect that kind of material behaviour, different tensile and compression tests were carried out. These tests were performed for different printing angles [0–90${}^{\circ }$], different layer heights (100 µm and 50 µm) and for different strain rates [10${}^{-4}$–10${}^{3}$ s${}^{-1}$], Table 1. This wide strain rate range will allow study of how a polymeric printed part behaves from a quasi-static situation (strain rates of $10^{-4}$ or $10^{-2}$ s${}^{-1}$) up to a scenario in which the printed part could be subjected to a dynamic load or even to impact (strain rates of $10^{3}$ s${}^{-1}$). In addition, the effects in curing time and temperature were also studied throughout the present work. The technical characteristics and the conditions used in each test will be shown in the following sections.

**Figure 1:**
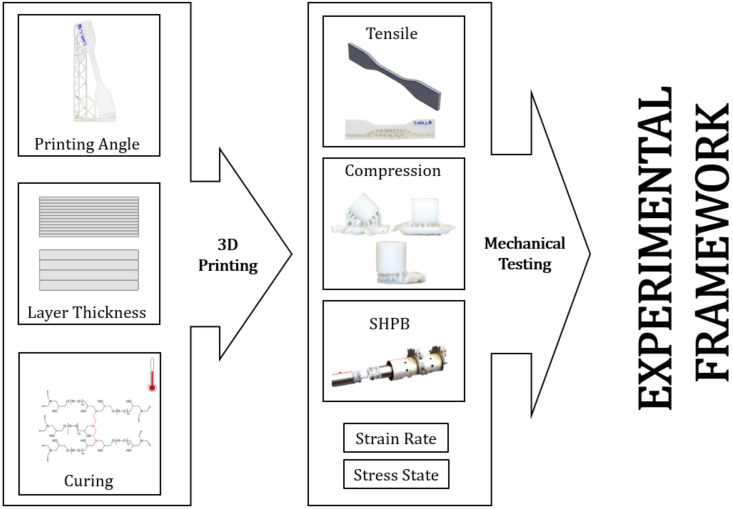
Experimental framework to study and characterize materials printed by SLA.

The first step in the experimental framework proposed in this paper has been done in Section 2 of this document in which the manufacturing process has been carefully analyzed and the driven manufacturing parameters arise from this analysis. The following step in the framework is to benchmark the mechanical performance of the printed material taking into account the stress state, the strain rate sensitivity and the aforementioned manufacturing parameters, a sketch summarizing the framework proposed is shown in Figure 1.

### 3.1. Specimen Geometries and Experimental Setups

#### 3.1.1. Tensile Test

The tensile tests were performed using specimens with the geometry *TYPE IV* recommended by the *ASTM D 638* standard, with a gauge length of 33.04±0.015 mm, a width of 6.15±0.028 mm and a thickness of 4.07±0.009 mm. This specimen was proposed because, due to its size, most 3D printers can print it.

**Figure 2:**
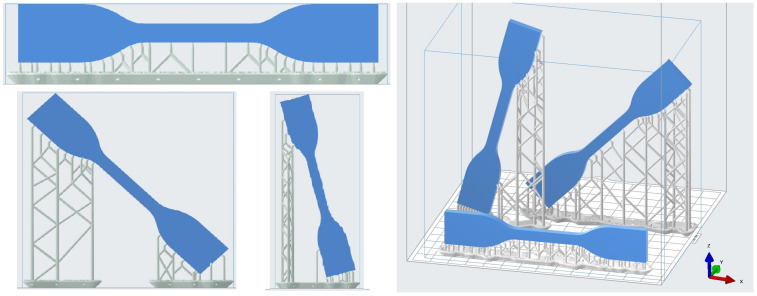
Printing angle variation in tensile coupons. The images correspond to the printing software simulations; the specimens are filled with blue and the supports in gray.

To study the influence of the printing angle (θ), the specimens were printed in 3 different orientations (0, 45 and $75^{\circ }$) as shown in Figure 2. The vertical structures that appear in the Figure 2 correspond to the supports or scaffolds that aid the correct growth of the sample and its attachment to the building bed, as already mentioned in the manufacturing steps. For repeatability reasons, the three different angles were printed in three different series or batches, resulting in a total of 9 specimens (three of each angle). In addition, each angle was printed using 2 different layer heights on the vertical axis *z*, (50 and 100 µm, respectively) to study the influence of that parameter on the mechanical properties of the printed material.

The tensile tests were carried out in servo-hydraulic equipment with a 10 kN load cell, following the recommendations contained in ISO527 and ASTMD638 standards [22,23]. The specimens were tested at different strain rates using two different cross head speeds: 0.02 and 0.2 mm/s. The axial strain was obtained directly from the machine displacement since the stiffness of the machine vastly exceeds the stiffness of tested samples. This fact was verified by the application of an external linear variable displacement transducer known as LVDT tooling that corroborated measurement values obtained from the machine, making them reliable enough for their use and subsequent treatment. In addition, a Digital Image Correlation (DIC) system was used to assure the correct performance of the test as well as to analyze the displacements and deformation of the specimens. In a first approach strain gauges were applied to the specimen but it was found that the adhesive used (cyanocrylate) weakened the material causing the premature breakage of the specimen and hence the measurement obtained was not correct, therefore a DIC system is recommended.

#### 3.1.2. Compression Test

**Figure 3:**
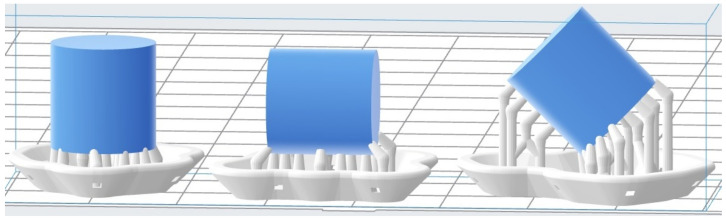
Printing angle variation for compression tests.

Compression tests were performed on specimens with a cylindrical geometry as recommended in *ASTM D 695* standard [24]. The specimen dimensions were 12 mm in height and in diameter. The influence of the printing angle (θ) was also considered in the compression tests, so the specimens were printed in 3 different orientations (0, 45 and $90^{\circ }$) as shown in Figure 3, where the mentioned supports or scaffolds can also be seen. As in the case of tensile specimens, the three different angles were printed in three different series or batches, for repeatability reasons, and with 2 different layer height on the vertical axis *z* (50 and 100 µm) to study the influence of that parameter on the mechanical properties of the printed material.

The compression tests were carried out in the same servo-hydraulic equipment used for the tensile tests. The tests were performed following the recommendations contained in ISO604/ASTMD695 standards. The specimens were tested at different strain rates using two cross head velocities: 0.02 and 0.2 mm/s, as in the tensile tests. The upper test compression tool has a hinge which avoids any shear loading due to small differences in parallelism between specimen faces and assure a correct performance of the test. In all tests, the upper and lower faces of the sample in contact with the test tool (tungsten carbide polished plates) were lubricated to ensure the correct application of the compression load and to reduce other loading phenomena that could appear.

**Figure 4:**
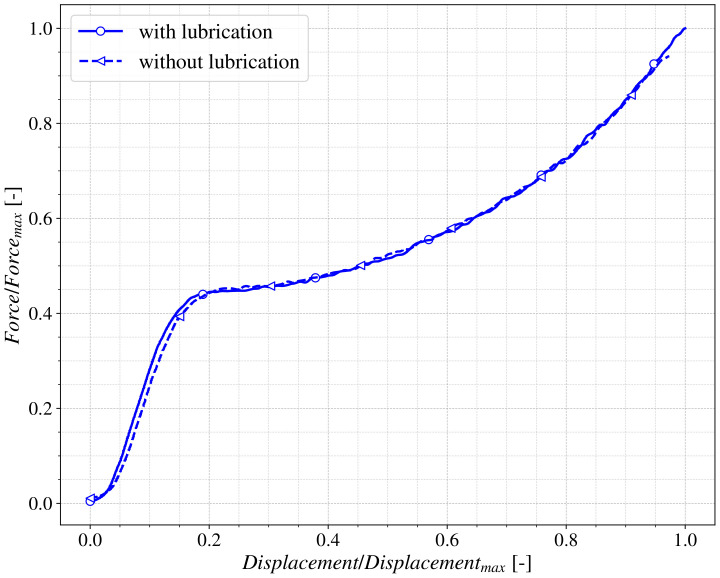
Lubrication effect on cylindrical samples in compression tests.

To verify that the lubrication applied did not have any noticeable effect on the behaviour of the material [25], it was considered interesting to make an additional study about the influence of the mentioned parameter. Compression tests were performed using specimens printed at the same printing angle (45${}^{\circ }$) and with the same layer height (100 µm) at different strain rates with and without lubrication. In addition, these specimens were printed in the same batch to avoid other effects that could interfere with the study of the isolated effect of lubrication. As can be observed in Figure 4 differences were less than a 1% in loading and displacement.

#### 3.1.3. Split Hopkinson Pressure Bar Compression Tests

The study of material behaviour under a dynamic regime was carried out on cylindrical specimens expressly prepared for a dynamic compression Hopkinson bar test (SHPB). The coupons were cylindrical with 6 and 10 mm diameter while the lengths of the cylinder were 10 and 12 mm to obtain different strain rates. As in the other cases, the specimens were also printed at different printing angles (0, 45 and $90^{\circ }$) and layer height (50 and 100 µm) in order to study its influence in the behaviour of the printed material.

Dynamic tests were carried out using a split Hopkinson pressure bar (SHPB). The system consists of a 22 mm diameter steel (F114) striker, input and output bars of 500, 2600 and 1500 mm length, respectively. The striker is fired, using pressurized air, at the input bar promoting the stress pulse, which travels inside the bar and hits the specimen located between the input and output bar, promoting the desired compression. At the interfaces between the loading bars and the specimen, the stress pulse separates into reflected and transmitted pulses at the input and output bars. Part of the pulse is transmitted to the output bar and reflected in the input bar due to the compression and mismatch impedance of the bar and the specimen. Impact striker velocity and strain induced in the input and output bars were measured using laser barriers and strain gauges in the incident bar. These measurements allow the obtaining, using 1D theory, of stress–strain curves of the material. Alignment of the bars was checked prior to testing using the impact velocity and the strain pulse magnitude and comparing the theoretical values. For more information about data reduction method and equilibrium assessment the reader is referred to [26,27]. By means of different specimen geometric values such as diameter and length it has been able to find and cover a wide range of deformation speeds (10${}^{3}$–10${}^{4}$ s${}^{-1}$). Since there is not an explicit standard for this type of test procedure nor material, it was decided to follow the recommendations contained in ISO 18872 [28] regarding dynamic characterization of polymers.

Table 1 summarizes the amount of characterization tests performed, considering all load application ranges.

## 4. Results and Discussion

**Figure 5:**
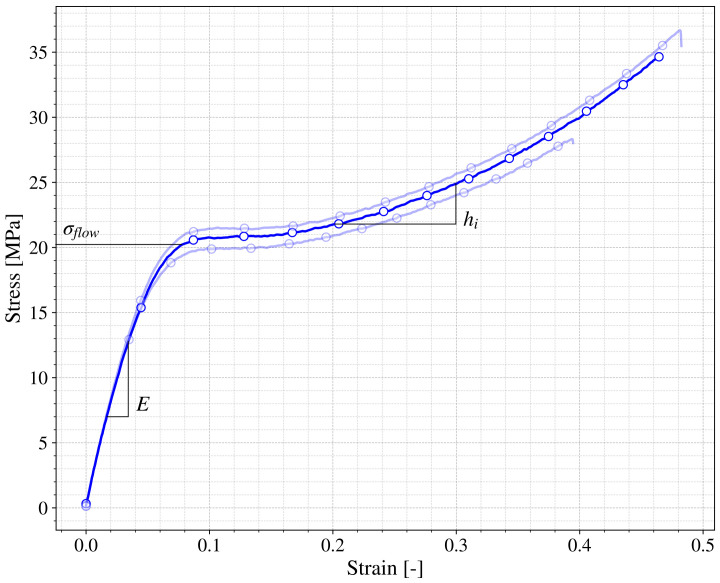
True stress versus true strain for a 45${}^{\circ }$ printed specimen in quasistatic tensile test.

The objective of this research is to propose an experimental framework or methodology to benchmark the materials obtained through a 3D printing SLA process. To show the feasibility of this framework the resin called *Durable* from Formlabs^®^ was selected. The representative true stress vs. true strain behavior of this resin is depicted in Figure 5 showing two trends according to the different slopes that appear in the curve. Three different curves tested under the same conditions are shown as an example of the repeatability of the test performed. The values of the true stress and strain were obtained from the force (F)-displacement (L) curves and the geometrical parameters of the specimens, as follows:

(1) $$\sigma =\frac{F}{A_{0}}\left(1+\frac{\Delta L}{L_{0}}\right)$$

(2) $$\varepsilon =\ln\left(1+\frac{\Delta L}{L_{0}}\right)$$

First a relatively stiff response corresponding to the elastic behavior can be seen, characterized by the Young modulus of the material (*E*). This trend finishes when a certain value of stress is reached ($\sigma_{flow}$) [29] in which the material starts to flow at a relative constant stress, with a certain hardening represented by the hardening modulus ($h_{i}$). This reference values $E,\sigma_{flow}$ and $h_{i}$ will be used to benchmark the different tests performed. It is important to note that this behavior is similar to polymers obtained through industrial methods that are not 3D printing [30].

### 4.1. Stress State Dependence

The understanding of the stress state dependence is a primary feature to take into account when additively manufactured specimens are employed in real applications. The scope of usefulness of a structure or a component is driven by its mechanical properties (mainly given by the basis material) and its behavior under complex stress state. To assess the printed material response under any kind of loading conditions, a simple study of tensile and compression can give some key points. For example, differences in the behavior of the material observed in these tests could lead to the conclusion that the material has a hydrostatic pressure dependence.

**Figure 6:**
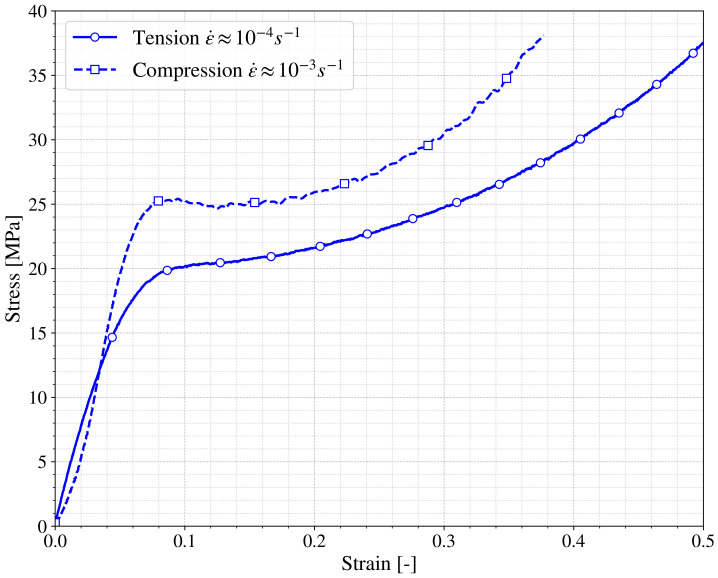
True stress vs. true strain of tensile and compression tests.

Figure 6 shows the behavior of the SLA specimens printed in the same angle and with the same layer height under tensile and compression. Both curves present a similar trend as the one previously presented in Figure 5 and characterized by $E,\sigma_{flow}$ and $h_{i}$. Nevertheless, the curves present a remarkable difference in the $\sigma_{flow}$ (around 20 MPa for tensile and 25 MPa in compression). This observation could lead to the conclusion that the printed material has pressure dependence and therefore a further investigation would be necessary for real applicability. However it has to be taken into account that although both tests have been done at the same cross-head velocity, the differences in the geometry of the specimens make the strain rate of both tests quite different and hence the increase of 25% of $\sigma_{flow}$ could be motivated by this fact, as will be discussed in the following section.

**Figure 7:**
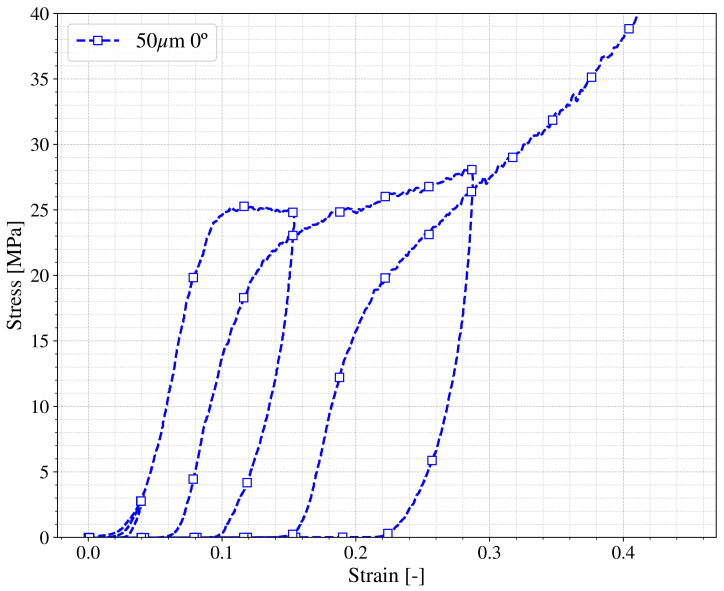
Loading and unloading compression behavior in True stress vs. true strain for a 0${}^{\circ }$ printed specimen.

The experimental framework that this work proposes also takes into account that the printed materials can be subjected to fatigue processes or other types of applications with loading and unloading states. Knowing how the printed material behaves in those situations is of great importance for designing purposes, therefore uni-axial tests of loading and unloading were performed imposing a series of cycles with increasing strain as can be seen in Figure 7. If the unloading curve changes with the maximum strain reached, the Mullins effect is presented in the printed material [18,29,31] and further investigation is needed. The material proposed for the present framework does not show significant Mullins effect since the unloading curves of Figure 7 at different strains (ε = 0.15 and 0.3) present similar slopes.

### 4.2. Strain Rate Dependence

**Figure 8:**
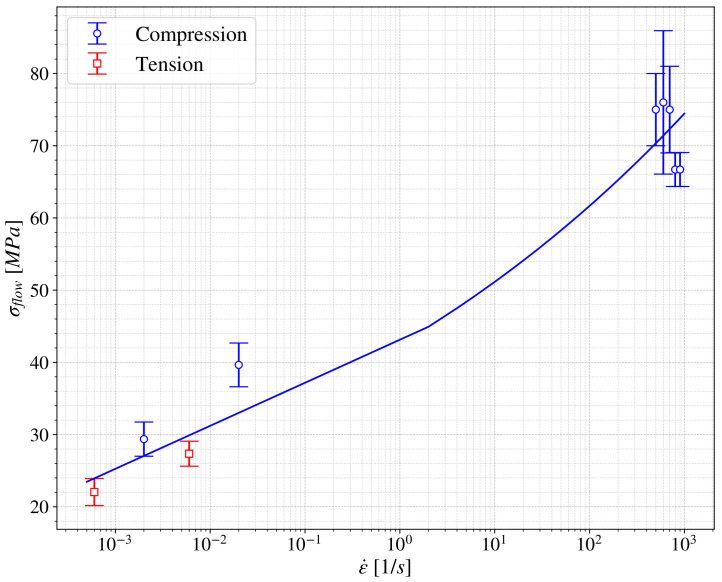
$\sigma_{flow}$ vs. $\dot{\varepsilon }$.

For dynamic applications it is important to unveil the strain rate dependence of the material, to focus on this: different cross head speeds, geometries and impact velocities were considered in quasistatic regime tests and Split Hopkinson Pressure Bar (SHPB) tests. The study focuses on the characteristic values of the printed material previously mentioned: *E*, $\sigma_{flow}$ and $h_{i}$. Once analyzed, the results regarding the mentioned parameters it can be concluded that the most important dependence observed in the printed material behavior is in $\sigma_{flow}$. Figure 8 shows this parameter versus the strain rate of the test. It is worth remembering that the tests at each strain rate were performed for the different printed angles and layer heights considered. Therefore, blue circles represent compression tests, red squares correspond to tensile tests and the error bar compiles the results of all the tests performed under each situation regarding printing angle and layer height. Scattering in the results could be considered low, although higher scattering as usual is observed in the tests performed at high strain rate ($10^{2}<\dot{\varepsilon }<10^{3}\;s^{-1}$). Despite this, it can be concluded that the printed material exhibits an important strain rate sensitivity; the value of $\sigma_{flow}$ raises from around 30 MPa up to almost 80 MPa in the range of the strain rate in study; the dependence shown could be estimated as follows:

(3) $$\sigma_{flow}=\sigma_{flow}^{initial}+C_{1}{\dot{\varepsilon }}^{C_{2}}$$

with $\sigma_{flow}^{initial}=2.5\;\mathrm{MPa}$, $C_{1}=40\;\mathrm{MPa}$ and $C_{2}=0.085$.

The observation regarding the dependence of the printed material with the strain rate is important because otherwise the differences shown previously in Figure 6 could be considered due to the dependence of the material to the hydrostatic pressure instead to strain rate (even in the quasistatic regime).

### 4.3. Influence of the Printing Parameters

The manufacturing process to obtain a 3D printing material may affect the behaviour of the final manufactured element due to the choice of different parameters that have to be taken into account in this kind of manufacturing technique, as shown in [32,33]. This behaviour dependence could be a barrier in the final use of these methodologies for industrial application. In this section, a proposal for the evaluation of the dependence of manufacturing in the behavior is proposed. The main driven parameters identified for the 3D printing technology considered are: printing orientation or angle, AM layer height and post-curing process.

#### 4.3.1. Influence of the Printing Angle

**Figure 9:**
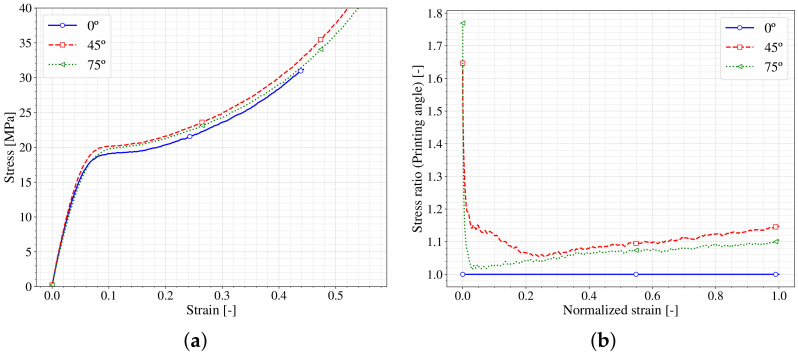
Printing angle influence in SLA process for quasistatic tensile tests. (**a**) σ vs. ε; (**b**) Ratio of differences.

It is known that the 3D printing process could be simplified to a 2D process stacking layer by layer. In Fused Deposition Manufacturing process different authors [34,35] show that the performance of the manufacturing structure or piece depends on the orientation of this stacking plane with respect to the load direction. Usually, the interface between the layers represents a weak zone for the material and thus should be taken into account. To study this effect in the SLA process, coupons at different printing angles were compared. Figure 9a shows the stress–strain curve obtained in quasistatic tensile tests for different coupons printed at different angles (0${}^{\circ }$, 45${}^{\circ }$and 75${}^{\circ }$), where all the curves correspond to the same strain rate. It is observed that the curves overlap showing almost no difference between them, therefore to deepen in the comparison the stress ratio between the curves versus the normalized strain is shown in Figure 9b. It can be seen that the highest stress ratio values appear at the beginning of the curves arising from the small errors made in the zero adjustment of the strain values. As soon as the normalized strain increases, the stress ratio decreases rapidly and then smoothly grows to values around 1.1, which corresponds to differences lower than 10% for the different printed angles cases. This ratio seems to be a good index to capture differences in the behavior of the printed material for several configuration tests. In fact, the same analysis was performed for different tests (tensile and compression), cross head velocities (0.02 and 0.2 mm/s) and layer height (50 and 100 µm) obtaining a similar trend which may confirm that the SLA printing process is almost insensitive to the effect of printing angles and therefore the printing material could be considered to be fairly isotropic for designing applications. The insensitivity to printing angle could be due to the post-curing process of the SLA printed material (Section 2) which homogenizes the material achieving an isotropic behaviour.

#### 4.3.2. Influence of the Layer Height

**Figure 10:**
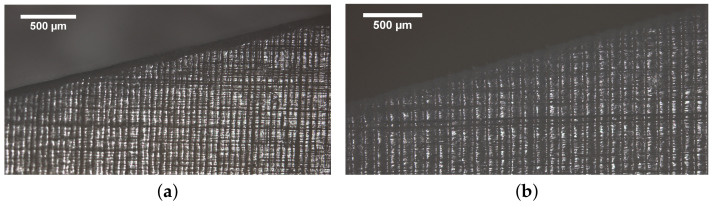
Effect of the Layer height in the surface finishing in samples printed at 75${}^{\circ }$. (**a**) 50 m; (**b**) 100 m.

**Figure 11:**
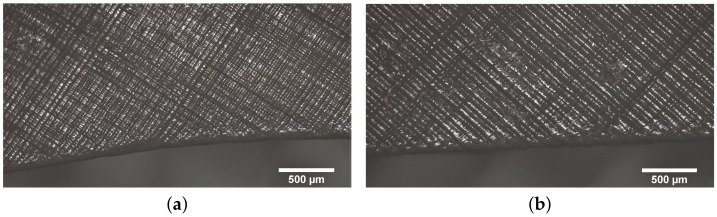
Effect of the Layer height in the surface finishing in samples printed at 45${}^{\circ }$. (**a**) 50 m; (**b**) 100 m.

One of the printing parameters that it is necessary to define to get a printed material or part is the layer height, as has already been mentioned. The layer height of the printed parts corresponds to the amount of material cured by the laser in each layer during the manufacturing process. Therefore, it can be directly related to the time spent in obtaining each part, so that a higher layer height means a lower printing time which could be positive. However, from a geometric point of view choosing a higher layer height implies getting a less accurate surface finishing. This should be taken into account for the zones in which the tolerance is a driven parameter. Figure 10 and Figure 11 show the differences in the surface finishing that appear in the cases with low and high layer height (50 and 100 µm) at different printed angles. The difference in the amount of material cured by the laser in each layer can also be observed, which could lead to differences in the behaviour of the printed parts.

**Figure 12:**
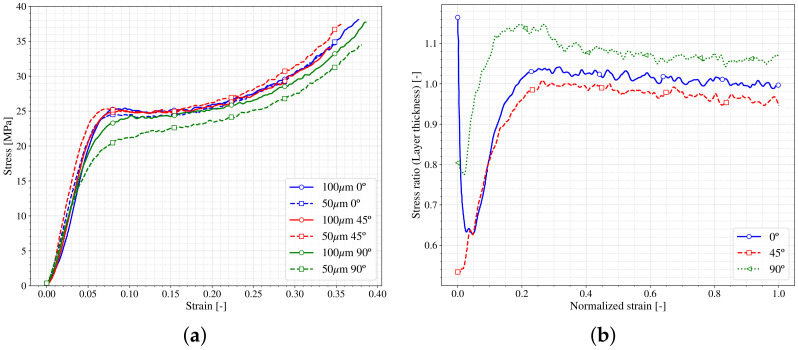
Layer height effects on stress-strain compression results. (**a**) σ vs. ε; (**b**) ratio of differences.

In terms of mechanical performance of the parts, Figure 12a shows the stress–strain curves obtained in quasistatic compression tests of different samples printed with different layer heights and printing angles. All the curves show the same trend previously seen in Figure 5; a first stiffer trend (*E*) up to reaching a stress value ($\sigma_{flow}$) from which appears a hardening ($h_{i}$) slope. No remarkable differences are observed regarding the different layer height of the samples. The values of the samples printed in 90${}^{\circ }$ at both layer height present slightly lower values of $\sigma_{flow}$, nevertheless this differences could be related to the inherent dispersion of the experimental setup. However, to perform a better comparison in terms of layer height the Figure 12b shows the stress ratio between the different layer height samples versus the normalized strain. The normalization has been performed comparing the stress-strain curves with different layer heights within each printing angle. For this, 50 µm curves have been taken as the reference and the ratio between both layer heights have been plotted as shown. As happened in Figure 9b, the differences are high at the beginning of the curves (small values of normalized strain) whereas when the value of the normalized strain increases, the stress ratio diminishes until reaching a value near one. These results allow a conclusion that the layer height may have a non-negligible influence in the mechanical behaviour of SLA printed parts at small strains (in the Elastic regime). However, when the strain increases, it could be said that the layer height has barely any influence on the behaviour of the specimens, for the ranges here considered. A deeper study should be done to unveil the role of the layer height on the mechanical performance of the material.

#### 4.3.3. Influence of the Curing

**Figure 13:**
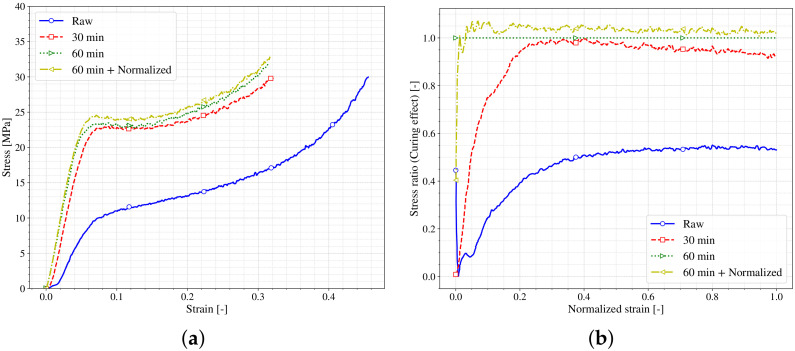
Post-Curing effect on stress-strain curves. (**a**) σ vs. ε; (**b**) ratio of differences.

As already mentioned, the last step in the stereolithography (SLA) manufacturing process corresponds to a post-curing of the samples. This step is done in an ultraviolet oven, which imposes on the manufactured piece a bath of ultraviolet in a controlled temperature space. Each resin requires different post-curing times, and the resin manufacturer suggests these times and temperatures. It has been observed experimentally that the degree of curing affects the performance of the resin [36]. In this section, a study of the effect of the time of ultraviolet/temperature exposition on the samples in the mechanical behavior is done. To this end, the behaviour under compression loading of samples subjected to different post-curing times are compared. Figure 13a shows the stress-strain curves of samples with different post-curing conditions; the raw specimen has not been subjected to any post-curing effect. The other samples were subjected to 30 or 60 min of post-curing both at 60 ${}^{\circ }$C. Additionally, the effect of cooling the sample from the post curing step inside the oven or at room temperature (outside the oven) is shown in the curves (60 min + Normalized and 60 min respectively). All the curves present similar trends: elastic (*E*), flow ($\sigma_{flow}$) and hardening ($h_{i}$), the values of these characteristic parameters differ from the raw specimen to the other specimens being much lower in the first case. It seems that the post-curing process is needed to finish the polymerization of the resin and hence achieve a better mechanical performance of the printed specimens. The specimens subjected to different times and conditions of post-curing present similar behaviour. To enhance this comparison, Figure 13b depicts the stress ratio of each sample with respect to the 60 min one (manufacturer recommendation) against the normalized strain. The biggest differences are obtained at the beginning of the strain, as happened previously, in which a small inconsistency in the zero setup could lead to big differences. However, these differences are reduced sharply in the 60 min + Normalized case, being able to conclude that the way of how the cooling of the specimen is performed (inside or outside the oven) does not affect the behaviour of the material. On the other hand, the sample with only 30 min of post-curing shows higher differences until reaching the 20% of the normalized strain. This fact indicates that the elastic (*E*) part of the curve presents higher dependence of this post-curing time than the other part of the behavior ($\sigma_{flow}$ and $h_{i}$), which remains more in-sensitive. Finally, it is observed that the raw material presents both differences in the elastic part *E* and in the $\sigma_{flow}$ (reaching a value around 50% from the reference) nevertheless this difference does not increase with the strain, so the hardening ($h_{i}$) process should be similar in both cases.

## 5. Conclusions

The present work proposes an experimental methodology framework to study and characterize materials printed by stereolithography (SLA) to clarify certain aspects that must be taken into account to broaden the use of this kind of materials. To this end, tensile and compression tests at different strain rates were carried out. To study the influence of certain printing parameters on the printed material behavior, samples with different printing angles ([0–90${}^{\circ }$]) and different printing resolution (100 µm and 50 µm layer height) were tested. In addition, the effects of curing time and temperature were studied. The testing specimens were manufactured in the non-professional SLA machine *Form 2* from Formlabs^®^ using resin called *Durable*; nevertheless, the experimental methodology proposed could be extended to any other resin. The following main conclusions can be drawn from the analysis and discussion of results:

- The experimental methodology proposed is capable of showing how the printed material behaves under different load situations such as tensile, compression or even dynamic conditions. This information could be very valuable for engineers and designers to develop final applications manufactured by SLA technology.
- The performance of dynamic tests is important to prove the influence of the strain rate on the printed material behaviour, and hence clarify the differences that may appear on the stress–strain curves of quasistatic tensile and compression tests. This helps to avoid mistakes in future applications of the printed material and hence with a good performance during product lifetime.
- The printing angle of the samples barely influences the mechanical behaviour. The test results show that the differences observed are lower than 10% for the different printed angles cases and hence the printing material obtained by SLA technique could be considered to be isotropic for designing applications. The post-curing process carried out in this kind of printing methodology could be responsible for achieving this isotropic behaviour.
- The results show that the mechanical behaviour of the SLA printed material may be affected, at small strains in the elastic regime, by the layer heights analyzed (50 and 100 µm). However, when the strain increases, it could be said that the layer height has barely any influence on the behaviour of the specimens, for the ranges here considered. This information is valuable for engineers and designers to assure better mechanical performance of the printed parts according to the strain range at which the printed element could be subjected. However, the layer height controls the finishing of the parts and hence these parameters should be taken into account in applications in which surface accuracy drive the design.
- The post-curing process is fundamental in the SLA manufacturing methodology to achieve the optimal behaviour of the printed material. A shorter post-curing time affects basically the elastic part of the behaviour whereas the hardening part is not affected. On the other hand, the time of cooling once the post-curing process has been carried out shows small influence in the final behaviour of the printed material.

The conclusions reached may contribute to the knowledge of this kind of printed material as well as be valuable for designers to broaden the use of the printing technology for final applications.

.

## Figures and Tables

**Table 1:** Summary of tests performed.

Test	Layer Height	$\dot{\varepsilon }\;\left[s^{-1}\right]$	θ [${}^{\circ }$]	ϕ [mm]	*L* [mm]	Specimen Per θ	Total Amount
Tensile	50 µm	∼10${}^{-4}$	0, 45, 75	ASTM D638 ${}^{1}$		2	15
		∼10${}^{-3}$	0, 45, 75	ASTM D638 ${}^{1}$		3	
	100 µm	∼10${}^{-4}$	0, 45, 75	ASTM D638 ${}^{1}$		2	15
		∼10${}^{-3}$	0, 45, 75	ASTM D638 ${}^{1}$		3	
Compress.	50 µm	∼10${}^{-3}$	0, 45, 90	12	12	3	18
		∼10${}^{-2}$	0, 45, 90	12	12	3	
	100 µm	∼10${}^{-3}$	0, 45, 90	12	12	3	18
		∼10${}^{-2}$	0 ${}^{2}$, 45, 90	12	12	3	
SHPB	50 µm	∼10${}^{3}$	0, 45, 90	10	10	3	18
	50 µm	∼10${}^{3}$	0, 45, 90	6	12	3	18
Total number of specimens							102

^1^ For tensile specimen dimension and geometries form ASTM D638 for specimen type IV has been adopted; ^2^ For compression specimens at 0${}^{\circ }$ printing angle and for a layer height of 100 µm four (4) more samples were printed for the curing effect study.

## Data Availability

The data presented in this study are available on request from the corresponding author.
